# Nicotinamide Riboside Enhances Mitochondrial Bioenergetics and Dopaminergic Signaling Independent of Neuron Survival in a Double-Hit Parkinson’s Model

**DOI:** 10.21203/rs.3.rs-9970859/v1

**Published:** 2026-06-19

**Authors:** Sukanya Saha, Chengbo Meng, Jie Dong, Jingheng Zhou, Sunny Wang, Amy Papaneri, Lixin Sun, Huaibin Cai, Guohong Cui

**Affiliations:** National Institute of Environmental Health Sciences; National Institute of Environmental Health Sciences; National Institute on Aging; National Institute of Environmental Health Sciences; National Institute of Environmental Health Sciences; National Institute of Environmental Health Sciences; National Institute on Aging; National Institute on Aging; National Institute of Environmental Health Sciences

**Keywords:** Parkinson’s disease, nicotinamide riboside, mitochondrial bioenergetics, dopamine release, α-synuclein, benomyl

## Abstract

**Background:**

Parkinson’s disease (PD) is characterized by progressive degeneration of *substantia nigra pars compacta* (SNc) dopaminergic (DA) neurons and the development of motor and non-motor impairments. Mitochondrial dysfunction, exacerbated by aging and environmental exposures, is a central contributor to PD pathogenesis. Nicotinamide riboside (NR), a dietary precursor of nicotinamide adenine dinucleotide (NAD^+^), enhances cellular bioenergetics and mitochondrial health, yet its translational potential for PD remains insufficiently defined.

**Methods:**

We used a “double-hit” PD mouse model combining A53T α-synuclein overexpression in SNc DA neurons with chronic dietary benomyl exposure. Mice received continuous NR supplementation in drinking water. Motor behavior was monitored longitudinally using open-field and rotarod assays. Striatal dopamine dynamics were quantified using genetically encoded fluorescent dopamine sensors to measure tonic and optogenetically evoked dopamine release. In vivo ATP/ADP ratios were measured in DA neurons and striatal spiny projection neurons (SPNs) using fiber photometry of the ratiometric sensor PercevalHR.

**Results:**

Chronic NR supplementation markedly improved motor performance in double-hit PD mice, despite failing to prevent SNc DA neuron degeneration. NR robustly increased both tonic and stimulus-evoked striatal dopamine release in control and PD mice. Additionally, NR elevated ATP/ADP ratios across multiple neuronal populations, indicating enhanced mitochondrial energetic capacity.

**Conclusions:**

NR supplementation enhances DA neurotransmission and mitochondrial bioenergetics in vivo, conferring functional benefits that occur independently of DA neuron survival. These findings identify metabolic augmentation via NR as a promising adjunctive strategy for mitigating PD-related functional deficits.

## Background

Parkinson’s disease (PD) is a progressive and debilitating neurodegenerative disorder characterized by both cardinal motor symptoms and a broad spectrum of non-motor impairments [1, 2]. Affecting millions worldwide, its global prevalence is projected to exceed ten million by 2030 [3–5]. Clinically, PD is primarily classified as a movement disorder due to early motor manifestations that arise from progressive degeneration of dopaminergic (DA) neurons in the nigrostriatal pathway [6, 7]. The age-dependent progression of PD and the limited efficacy of current disease-modifying therapies contribute to significant morbidity [8–10]. Although the etiology of PD remains incompletely understood, it is widely believed to result from a complex interplay between genetic susceptibility and environmental toxin exposure [11].

A pathological hallmark consistently associated with PD is the abnormal aggregation of α-synuclein into Lewy bodies [12, 13]. Mutations in the α-synuclein gene increase the protein’s tendency to misfold and aggregate, disrupt its normal physiological functions, and are strongly linked to familial forms of PD [14]. These mutations, together with impairments in protein degradation pathways and environmental stressors such as oxidative damage, drive the accumulation of α-synuclein and the formation of Lewy pathology [14–18]. To investigate the mechanisms associated with mutant α-synuclein, we developed a mouse model targeting the *substantia nigra pars compacta* (SNc). This was achieved by stereotaxic bilateral delivery of adeno-associated viruses (AAVs) engineered to express the A53T α-synuclein mutation, inducing localized protein aggregation within the SNc.

Mounting evidence also implicates environmental toxins, including pesticides and fungicides, in PD etiopathogenesis [19, 20]. Epidemiological studies have reported that high exposure to benomyl, a benzimidazole fungicide containing a carbamate moiety, is associated with increased PD risk [21, 22]. Benomyl inhibits aldehyde dehydrogenase (ALDH), a key enzyme in dopamine metabolism responsible for detoxifying 3,4-dihydroxyphenylacetaldehyde (DOPAL), a cytotoxic dopamine metabolite generated by monoamine oxidase [21, 23]. ALDH inhibition is expected to cause DOPAL accumulation, which can promote neuronal injury [24]. Thus, benomyl exposure may increase PD risk and accelerate disease onset. In this study, benomyl-contaminated chow was used as the environmental insult in a combined “double-hit” PD mouse model.

Nicotinamide adenine dinucleotide (NAD^+^) plays a central role in cellular energy metabolism, acting as an electron carrier that facilitates nutrient conversion into ATP. NAD^+^ also supports dopamine metabolism by enhancing ALDH activity and promoting conversion of DOPAL into its non-toxic metabolite, DOPAC. Nicotinamide riboside (NR), a vitamin B3 derivative, is considered one of the most efficient NAD^+^ precursors [25–29]. NAD^+^ levels decline with age and in several neurodegenerative conditions, and NR supplementation has been shown to improve metabolic health, promote detoxification, and support cellular resilience [28–31]. Despite these reported benefits, it remains unknown whether NR can reduce PD risk or ameliorate PD-related dysfunction, particularly in the context of interacting genetic and environmental stressors.

The overarching goal of this study was to determine whether NR supplementation can mitigate PD-related deficits in our double-hit mouse model, which combines A53T α-synuclein overexpression with chronic benomyl exposure. To this end, we conducted longitudinal behavioral assessments, *in vivo* fiber photometry to monitor cellular energy dynamics and dopamine release, and histopathological analyses of DA neuronal integrity.

## Materials and Methods

### Mice

All animal procedures were approved by the Institutional Animal Care and Use Committee of the National Institute of Environmental Health Sciences (NIEHS). Experiments were performed using male and female mice aged 3–10 months. Adult *Dat*^*IREScre/+*^ mice (Jackson Laboratory, #006660) were obtained from the Jackson Laboratory (JAX). Transgenic D1-Cre (MMRRC_036916-UCD) and A2A-Cre (MMRRC_036158-UCD) mice were obtained from the Mutant Mouse Resource Research Centers (MMRRC). Experimental *Dat*^*IREScre/+*^ mice were generated by breeding *Dat*^*IREScre/+*^ homozygous transgenic mice with C57BL/6J mice (JAX, #000664). Experimental D1-Cre and A2A-Cre mice were generated by breeding the D1-Cre and A2A-Cre hemizygous BAC transgenic mice with C57BL/6J mice.

Mice were singly housed after 9–10 weeks of age in a standard 12-h light/dark cycle. PD model mice had ad libitum access to benomyl-treated chow for five weeks and to NR-supplemented drinking water for 21 weeks. Outside these time windows, all mice had ad libitum access to standard chow and water.

#### “Double-hit” PD model development

To induce α-synuclein pathology, *Dat* ^*IREScre/+*^ mice underwent bilateral stereotaxic injections of AAV1-Ef1a-DIO-SNCA-A53T-hGHpA (AAV-A53T) or Cre-dependent mCherry control virus (AAV1-mCherry; pAAV-hSyn-DIO-mCherry, Addgene #50459) into the SNc (coordinates from bregma: AP − 3.10 mm, ML ± 1.50 mm; DV − 3.75 mm). After ~5 weeks of expression, mice received a second environmental insult with benomyl-containing chow for five weeks (500 mg/kg chow). Benomyl chow was prepared by dissolving 500 mg benomyl in 150 ml corn oil and mixing with 1 kg NIH-31 chow overnight; control chow was treated with corn oil alone. Food was replenished twice weekly. Body weight and food consumption were monitored to assess taste aversion; no differences from regular chow were observed.

### Viral vectors and AAV construction

AAV1-Ef1a-DIO-SNCA-A53T-hGHpA (AAV-A53T), packaged with AAV1 capsid, was produced at titers of 1.3 × 10^13^–2 × 10^14^ gc/ml. Cre-dependent mCherry control virus (AAV-mCherry; pAAV-hSyn-DIO-mCherry, Addgene #50459) was generated by the NIEHS Viral Vector Core using AAV9 capsid (1.7 × 10^12^ gc/ml) [32]. AAV9-hSyn-DIO-PercevalHR (1 × 10^13^ gc/ml), enabling Cre-dependent expression of the ATP/ADP sensor, was produced by WZ Biosciences (PercevalHR plasmid from Addgene #49082) [33]. The dopamine sensor construct (pAAV-hSyn-mRuby2-GSG-P2A-DA2m-WPRE-pA) was generated using pAAV-hSyn1-mRuby2-P2A-GCaMP6f (Addgene #50943) and pAAV-hSyn-GRAB_DA2m (Addgene #140553). Restriction enzymes EcoRI and HindIII and In-Fusion HD Cloning (Takara/Clontech) were used. The resulting AAV9-hSyn-mRuby2-DA2m was packaged by the NIEHS Viral Vector Core (2 × 10^13^ gc/ml) [34, 35]. ChrimsonR was expressed using pAAV-Syn-FLEX-rc[ChrimsonR-tdTomato] (Addgene #62723-AAV5, 2 × 10^13^ gc/ml).[36].

### Viral Expression Procedures

**F**or the PD model, AAV1-DIO-SNCA-A53T or AAV9-DIO-mCherry was bilaterally injected into the SNc of DAT-Cre mice using stereotaxic coordinates described above.

For ATP/ADP measurements, AAV9-DIO-PercevalHR was injected into:

- DLS (AP + 0.50 mm, ML ± 2.30 mm, DV − 2.75 mm) of D1- and A2A-Cre mice
- SNc of DAT-Cre mice (same coordinates as AAV-A53T)

For dopamine-release measurements, AAV9-mRuby2-DA2m was injected into the DLS and AAV5-FLEX-ChrimsonR-tdTomato into the SNc.

All injections were bilateral, delivering 500 nl per SNc site and 1 μl per DLS site at 0.1 μl/min using a Hamilton syringe. Needles remained in place for 10 min before withdrawal.

### NR administration

NR (nicotinamide riboside chloride; ChromaDex, 98.7–98.9% purity) was delivered chronically via drinking water at 12 mM. For acute dosing, NR was freshly dissolved in sterile saline daily and administered intraperitoneally at 1000 mg/kg, twice daily (9:00 and 20:00) for five days.

### Optical fiber implantation for fiber photometry

For dopamine recordings, DAT-Cre mice were implanted with DLS fibers 4–5 weeks after AAV injections. Burr holes were drilled bilaterally over the DLS of DAT-Cre mice. Optical fibers were gradually lowered using 200 μm steps until emission spectra were detected, then lowered in 50 μm steps until fluorescence plateaued. The final coordinates of implanted fibers were (from bregma) AP 0.5 mm, ML +−2.4 mm, DV ~ −2.2 mm for DLS. Fibers were secured with Metabond.

For PercevalHR recording, mice underwent fiber implantation two months after AAV injection. Burr holes were drilled bilaterally over the SNc (DAT-Cre mice) or DLS (D1-/A2A-Cre mice). The fibers were implanted at the final coordinates (from bregma) of AP −3.1 mm, ML +−1.75 mm, DV −3.7~−4 mm for SNc; and AP 0.5 mm, ML +−2.4 mm, DV ~ −2.2 mm for DLS, and were secured with Metabond. Mice recovered for two weeks before recordings.

### Spectrally resolved fiber photometry

Spectrally resolved fiber photometry recordings for dopamine sensor GRAB_DA2m_ and ATP/ADP ratio sensor PercevalHR were performed in awake, freely moving mice as described previously[37, 38]. The recordings were carried out in an open-top darkened mouse operant chamber (21.6 ×17.8 ×12.7 cm, Med Associates) housed in a sound attenuating box. To record tonic and optogenetically evoked DA levels in the DLS, 473 nm laser (45–55 μW, measured at the end of the fiber patch cable) was used to excite GRAB_DA2m_ and mRuby2. Emitted photons from the fluorophores were collected by a spectrometer (OceanFX, Ocean Optics) at 20-Hz with 25-ms integration time for each time frame. For optical stimulation of ChrimsonR, a 15-s train of 561-nm laser pulses (4.8–5.1 mW measured at the end of the fiber patch cable) was delivered at 20 Hz with 10-ms pulse width through the same implanted optical fiber for fiber photometry recordings. Each 10-ms pulse was timed to occur within the 25-ms interval separating adjacent 25-ms spectrometer acquisition windows, thereby avoiding interference with the fiber photometry recording. Behavior was recorded simultaneously via a TTL-triggered camera (FLIR Grasshopper3). Linear spectral unmixing was performed on the acquired spectra using custom R scripts (available at: https://www.niehs.nih.gov/research/atniehs/labs/ln/pi/iv/tools/index.cfm) to generate two coefficients that represent the fluorescence intensity of GRAB_DA2m_ and mRuby2. The fluorescence ratio of GRAB_DA2m_ /mRuby2 was used to report the DA levels.

The dopamine release recordings in control mice lasted 21 minutes. Tonic DA levels before and after NR/saline were quantified at 50–55 s and 1,135–1,140 s, respectively. The first and last optogenetically evoked DA responses were averaged over 70–75 s and 1,150–1,155 s, respectively. The dopamine release recordings in the “double-hit” PD mice lasted 35 minutes. Tonic DA levels were measured during 2-s windows preceding the 3rd, 13th, and final stimulations (averaged over 293–294 s, 1,493–1,494 s, and 1,973–1,974 s, respectively). The corresponding optogenetically evoked DA responses were averaged over 308–309 s, 1,513–1,514 s, and 1,993–1,994 s, respectively.

To measure ATP/ADP ratios, PercevalHR fluorescence was recorded using alternating 447-nm and 488-nm laser excitation (45–55 μW, measured at the fiber tip) at 50 Hz, controlled by a two-channel pulse generator. Fluorescence spectra were acquired at 50 Hz with an 11-ms integration time using a spectrometer (QEPro, Ocean Optics). The ATP/ADP ratio was calculated as the ratio of PercevalHR fluorescence (total photon counts between 500 and 550 nm) under 488-nm excitation to that under 447-nm excitation[33]. Mitochondrial energetics recordings lasted 30 minutes. Baseline ATP/ADP ratios and responses to NR or saline i.p. injection were quantified over 0.5-s windows at 5 minutes (baseline) and 25 minutes (post-injection) during the recording.

### Behavioral tests

#### General Procedures

Mice acclimated for 45 min in the behavioral room. Experiments were conducted between 9:00 and 14:00. Mice were randomly assigned to groups based on baseline performance. Experimenters were blinded to treatment.

#### Open-Field Test

Spontaneous locomotion was measured in a 27.31 × 27.31 × 20.32 cm chamber equipped with infrared sensors (Med Associates). Mice explored freely for 30 min. Total distance, ambulatory counts, stereotypic counts, and vertical counts were quantified using Med Activity software.

#### Rotarod Test

Motor coordination was assessed using an accelerating rotarod (4–40 rpm; Maze Engineers model 5703). Mice performed three trials/day for three consecutive days at multiple experimental timepoints. Latency to fall (or passive rotation) was measured, and the mean of three trials used for analysis. Mice were trained one day before the initial assessment. Rest intervals between trials were ~ 20 min.

#### Immunohistochemistry

Mice were euthanized with pentobarbital and perfused with PBS followed by 4% PFA. Brains were post-fixed for 24 h, cryoprotected in 30% sucrose, and sectioned coronally at 35 μm.

Sections were blocked with 10% normal donkey serum and 0.1% Triton X-100. Primary antibodies included:

- anti-TH (1:1000, Millipore AB152)
- anti-A53T α-synuclein (1:500, Santa Cruz sc-12767)
- anti-RFP (1:1000, Abcam ab62341)

Secondary antibodies were Alexa Fluor–conjugated donkey antibodies (Invitrogen). Sections were mounted in VECTASHIELD with DAPI and imaged on a Zeiss LSM 780 confocal microscope.

For other experiments, goat serum was used, and antibodies against TH, GFP, mRuby2, or tdTomato were applied as appropriate.

#### Cell counting of TH + DA neurons

TH^+^ neurons were quantified using AIVIA 10 software. Eight sections per mouse were analyzed using pixel and object classifiers to identify TH^+^ cells. Automated results were verified manually with overlays, and false positives or missed cells were corrected.

#### Quantification of TH^+^ terminal density

DLS TH immunoreactivity was quantified using FIJI (ImageJ). RGB images were split, DAB intensity extracted, converted to inverted grayscale, and thresholded (45–255). Mean optical density (OD) was calibrated using a density standard, and background OD was subtracted. Six sections per mouse were analyzed.

#### Statistical analysis

GraphPad Prism 10 was used for statistical analyses. Tests included one-way and two-way ANOVA with appropriate post-hoc comparisons, repeated measures two-way ANOVA, and paired or unpaired t-tests. Sample sizes, P values, and mean ± SEM are reported in the text or figure legends.

## Results

### NR improves motor function in the “double-hit” PD mice

**Figure 1:**
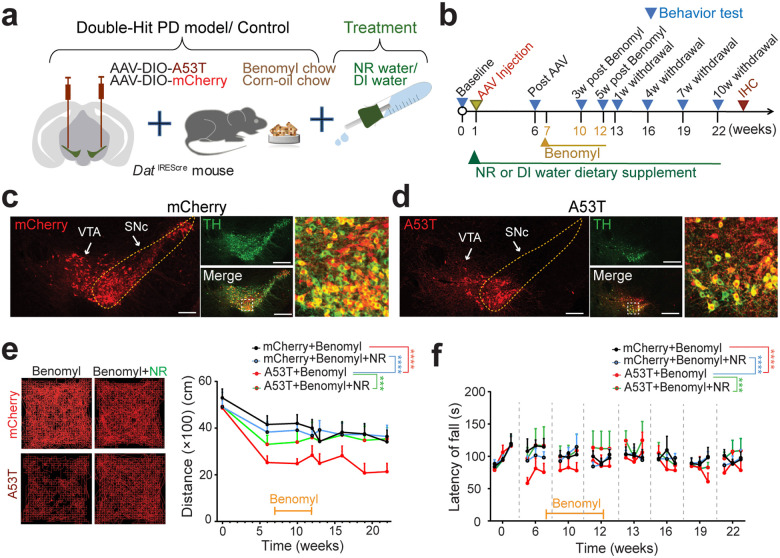
NR improves motor function in the “double-hit” PD mouse model **(a–b)** Schematic representation of the “double-hit” PD paradigm. *Dat* ^IREScre/+^ mice received bilateral SNc injections of AAV1-Ef1a-DIO-SNCA-A53T (A53T) or AAV9-hSyn-DIO-mCherry (control), followed six weeks later by benomyl-contaminated chow (500 mg/kg) for five weeks. NR supplementation (12 mM in drinking water) began on the day of AAV injection and continued throughout the study. Behavioral testing was performed from baseline to 21 weeks post-injection. **(c–d)** Immunostaining confirmed specific expression of mCherry or A53T α-synuclein in SNc DA neurons. Scale bar: 200 μm. **(e)** Open-field analysis showing reduced locomotion in double-hit mice relative to benomyl-treated controls, and significant rescue of ambulatory distance by NR at all post-treatment time points. Data are presented as mean ± SEM from 8 mice/group (*n*=8). Significance (*P < 0.05) was determined by two-way ANOVA followed by Tukey’s multiple comparisons test. **(f)** Rotarod performance demonstrating impaired motor coordination in double-hit mice and partialrescue by NR, with increased latency to fall. NR had no effect on control mice. Data are presented as mean ± SEM from 8 mice/group (*n*=8). Significance (*P < 0.05) was determined by two-way ANOVA followed by Tukey’s multiple comparisons test.

To generate a double-hit PD mouse model, *Dat*^IREScre/+^ mice received bilaterally injections of Cre-dependent AAV1-Ef1a-DIO-SNCA-A53T (A53T) or AAV9-hSyn-DIO-mCherry (control) into the SNc. Six weeks later, mice were exposed to benomyl-contaminated chow (500 mg/kg) for five weeks (Fig. 1a, b). Immunostaining confirmed robust and specific expression of mCherry or A53T α-synuclein in DA neurons (Fig. 1c, d). To assess whether NR could alter behavioral deficits or pathology in this model, NR supplementation (12 mM in drinking water) began on the same day as the AAV injections and continued throughout the study. Behavioral analyses were performed from one week prior to AAV injection (baseline) to 21 weeks post-injection (Fig. 1b).

In the open-field test, “double-hit” mice (A53T + benomyl) exhibited markedly reduced locomotion compared with benomyl-exposed controls. NR supplementation significantly increased ambulatory distance and various locomotory counts in the double-hit group across all measured time points (Fig. 1e; Supplementary Fig. 1a–c). Consistent with these findings, rotarod testing revealed impaired motor coordination in the double-hit mice, which were partially rescued by NR, as indicated by longer latency to fall relative to untreated double-hit animals (Fig. 1f). NR had no measurable effect on the performance of control mice. Exposure to benomyl contaminated chow alone did not alter any changes in motor behavior (Supplementary Fig. 2a-f).

Together, these results demonstrate that NR supplementation robustly ameliorates motor impairments in the double-hit PD model.

#### NR does not prevent the loss of DA neurons or their striatal innervation.

**Figure 2:**
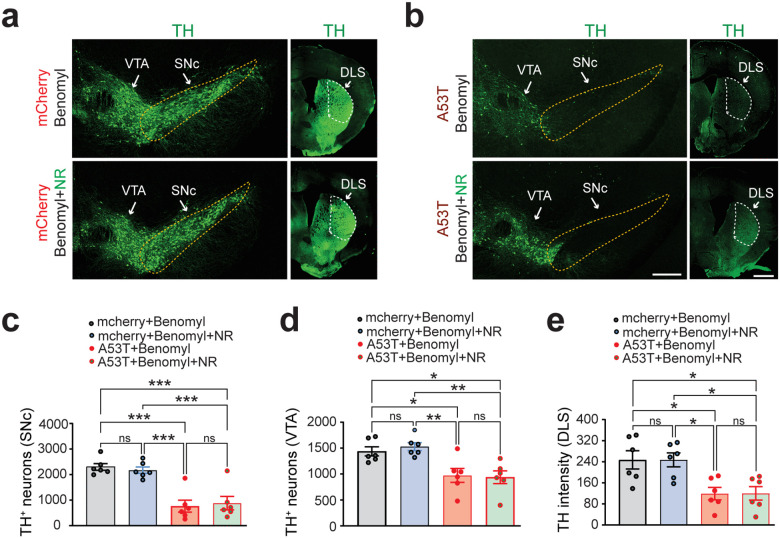
NR does not prevent DA neuron or terminal loss **(a–b)** Representative images of TH-positive neurons in the SNc and VTA and TH-positive terminals in the DLS. **(c–e)** Quantification showing >50% loss of SNc DA neurons, ~30% loss in the VTA, and >50% reduction of DLS dopaminergic terminals in double-hit mice, unaffected by NR. Data are presented as mean ± SEM from 6 mice/group (*n*=6). Significance (*P < 0.05) was determined by two-way ANOVA followed by Tukey’s multiple comparisons test.

In PD mice, histological analyses revealed > 50% loss of DA neurons in the SNc and ~ 30% loss in the ventral tegmental area (VTA), as well as > 50% reductions in DA terminals within the dorsolateral striatum (DLS), compared to the littermate control mice. These deficits were present in the double-hit model regardless of NR supplementation (Fig. 2a–e, Supplementary Fig. 1d–g). The A53T expression was comparable between the PD mice and NR supplemented PD mice (Supplementary Fig. 1h-j). Similar outcomes were observed when assessing the PD vulnerable aldehyde dehydrogenase 1A1-positive (ALDH1A1^+^) DA neuron subtype [39] and their projections, which were significantly reduced independent of NR treatment in the PD mice model (Supplementary Fig. 3a-d).

### NR acutely enhances striatal dopamine release

**Figure 3:**
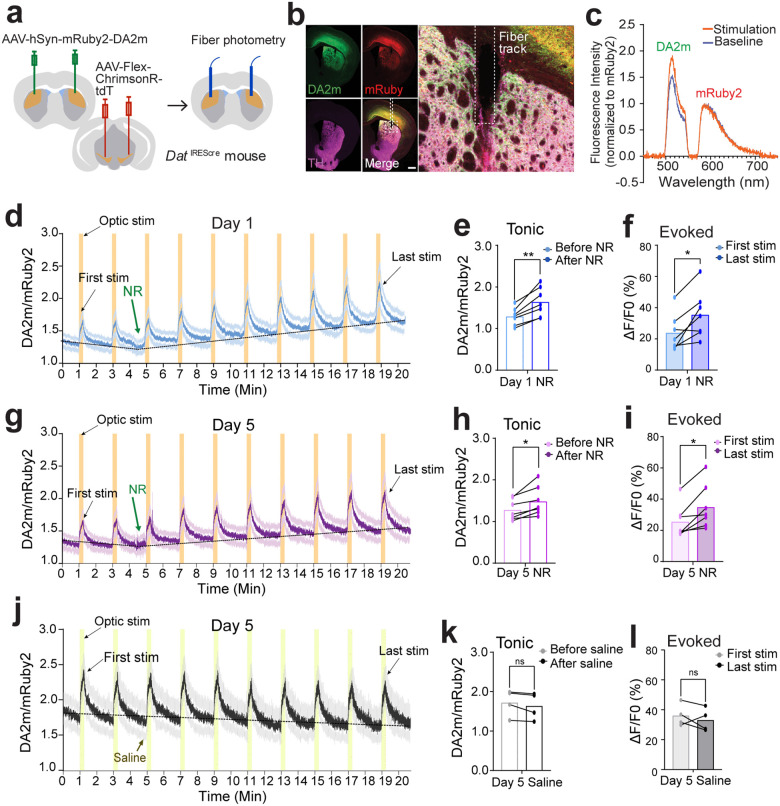
NR acutely enhances striatal dopamine release in vivo **(a)** Workflow for expressing DA2m in the DLS and ChrimsonR in SNc DA neurons. **(b)** Figure showing co-expression of GRAB_DA2m_ (green) and mRuby2 (red) in the DLS of control *Dat* IREScre/+ mice. **(c)** Representative emission spectra of DA2m and mRuby2 measured at the baseline and after optogenetic stimulation by Chrimson R. **(d–i)** NR produced rapid increases in tonic dopamine levels (d,e,g,h) and robust enhancement of optogenetically evoked dopamine release (f,i) on both day 1 and day 5, respectively. The tonic response was larger after the first NR dose (day 1). Data are presented as mean ± SEM 7 hemispheres of 5 mice/group. (*n*=7). Significance (*P < 0.05) was determined by paired t-test. **(j-l)** Saline injections produced no detectable changes in tonic or evoked dopamine release. NR acutely potentiates striatal dopamine release across repeated administrations. Data are presented as mean ± SEM from 4 hemispheres of 3 mice /group(*n*=4). Significance (*P < 0.05) was determined by paired t-test.

Since NR supplementation robustly ameliorates motor impairments in the double-hit PD model (Fig. 1), we next examined whether NR also modulates dopamine release *in vivo*. To measure NR-induced changes in dopamine dynamics, we co-expressed the green fluorescent dopamine sensor GRAB_DA2m_ and the red fluorescent control reporter mRuby2 (AAV9-hSyn-mRuby2-DA2m) in the DLS, while driving optogenetic stimulation of nigrostriatal DA axons by expressing the optogenetic actuator ChrimsonR (AAV5-Syn-FLEX-rc [ChrimsonR-tdTomato]) in the SNc of *Dat*^IREScre/+^ mice (Fig. 3a, b). Spectrally resolved fiber photometry [37] was then used to monitor tonic and optogenetically evoked dopamine levels following intraperitoneal injections of NR or saline.(Fig. 3c).

Acute NR administration (1000 mg/kg, i.p.) elicited a rapid increase in extracellular dopamine levels (Fig. 3d–i). This enhancement was observed on both day 1 and day 5 of NR treatment (Fig. 3d, g). Notably, the first NR dose produced a larger increase in tonic dopamine on day 1 compared to the day 5 response (Fig. 3e, h), whereas NR-evoked changes in dopamine release remained robust across both time points (Fig. 3f, i). In contrast, saline injections for 5 days produced no measurable change in either tonic or evoked dopamine release (Fig. 3j–l). These findings indicate that NR acutely potentiates striatal dopamine release *in vivo* in control mice.

### NR enhances striatal dopamine release in “double-hit” PD mice

**Figure 4:**
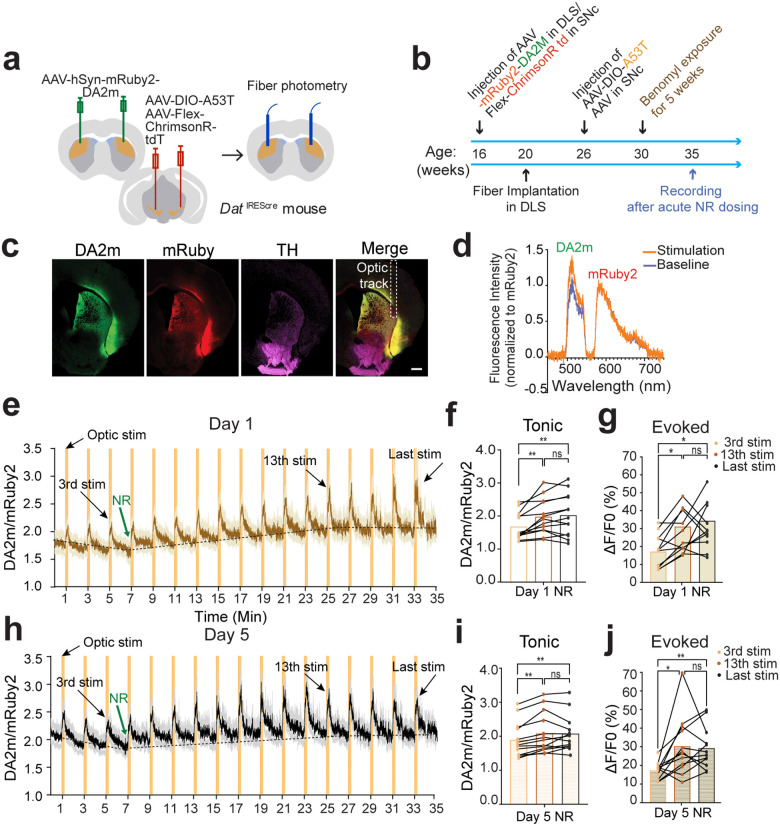
NR enhances dopamine release in “double-hit” PD mice **(a)** Workflow for expressing DA2m in the DLS and ChrimsonR in SNc DA neurons, followed by AAV-SNCA-A53T injection and benomyl exposure to generate the double-hit model. **(b)** Fiber photometry experimental timeline used to measure tonic and evoked dopamine release beforeand after acute NR administration. (c) Figure showing co-expression of GRAB_DA2m_ (green), mRuby2 (red) and TH (magenta) in the DLS **(d)** Representative emission spectra of DA2m and mRuby2 measured at the baseline and after opticalstimulation with ChrimsonR. **(e–j)** In double-hit mice, NR injections (1000 mg/kg, i.p.) produced immediate and robust increases in tonic (f, i) and evoked (g, j) dopamine release on day 1 (e-g) and day 5 (h-j). Responses plateaued after ~13 stimulations (~18 min post-injection), as shown in summary plots (g,j). Baseline tonic dopamine levels were elevated on day 5 prior to NR injection (e,h), suggesting cumulative enhancement of dopaminergic tone with repeated NR exposure. Data presented as mean ± SEM from 13 hemispheres of 8 mice/group (*n*=13). Significance (*P < 0.05) was determined by RM one-way ANOVA followed by Tukey’s multiple comparisons test.

To assess whether NR enhances dopamine release in the “double-hit” PD model, we first co-expressed GRAB_DA2m_ and mRuby2 in the DLS and ChrimsonR bilaterally in the SNc of *Dat* ^IREScre/+^ mice. Then, after allowing sufficient time for expression, we injected AAV1-Ef1a-DIO-SNCA-A53T into the SNc to induce α-synuclein (A53T) overexpression and subsequently implanted optical fibers above the DLS (for recording and optogenetic stimulation). Mice were then exposed to benomyl chow to complete the “double-hit” paradigm (Fig. 4a–c).

Spectrally resolved fiber photometry was used to monitor striatal tonic and optogenetically evoked dopamine release before and after acute NR administration (Fig. 4d). In the PD mice, NR injections (1000 mg/kg, i.p.) triggered immediate and robust increases in both tonic and evoked DA release on day 1 and day 5 of treatment (Fig. 4e–j). The NR-induced enhancement of dopamine signals approached a plateau after approximately the 13th stimulation (~ 18 minutes post NRinjection), as reflected in the summary bar graphs (Fig. 4f, g, i, j).

Notably, prior to NR administration on day 5, the baseline tonic dopamine level was already elevated compared to day 1 (Fig. 4e, f, h, i), suggesting that repeated NR exposure may confer a cumulative enhancement of dopaminergic tone in the “double-hit” PD model.

### NR restores bioenergetic capacity in DA neurons during PD-relevant stress

**Figure 5:**
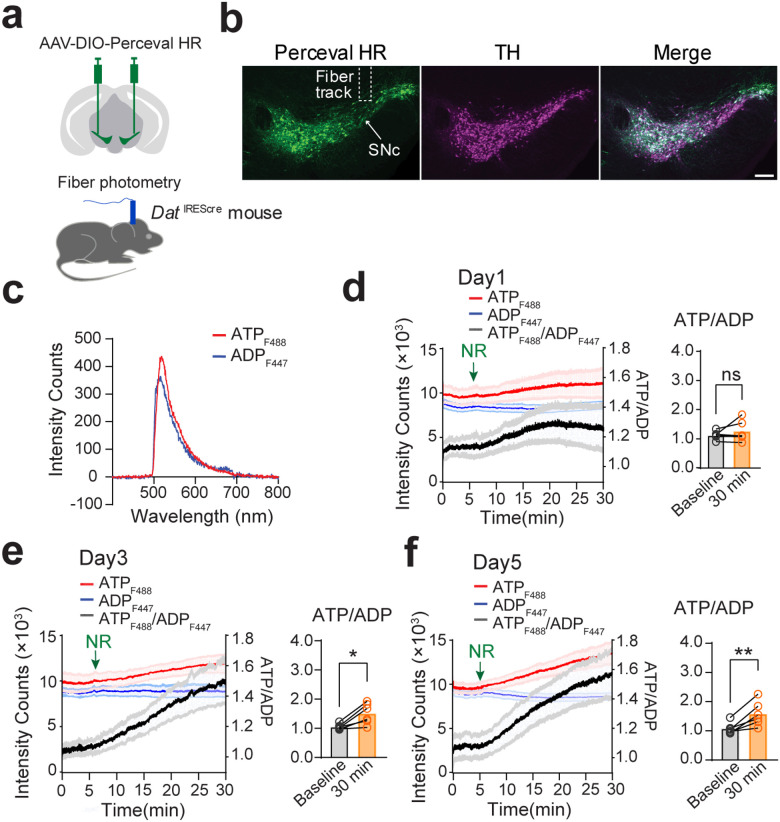
NR restores mitochondrial bioenergetic capacity in SNc DA neurons **(a–b)** Expression of the ATP/ADP sensor PercevalHR in SNc DA neurons following bilateral AAV9-hSyn-DIO-PercevalHR injection into Dat ^IREScre/+^ mice. **(c)** Representative PercevalHR emission spectra excited by 488 nm (for detecting ATP) and 447 nm (for detecting ADP) lasers using spectrally resolved fiber photometry *in vivo*. **(d–f)** ATP/ADP dynamics measured over a 5-day NR treatment regimen (twice daily; 1000 mg/kg, i.p.). On day 1, NR produced no detectable change in ATP/ADP ratio (d). On days 3 and 5, NR induced a significant increase in ATP levels and a decrease in ADP, resulting in elevated ATP/ADP ratios. Repeated NR exposure enhances mitochondrial energetic output in SNc DA neurons. Data are presented as mean ± SEM from 6 hemispheres of 4 mice/group (*n*=6). Significance (*P < 0.05) was determined by paired t-test.

To investigate the mechanism by which NR supplementation alleviates motor deficits and improve dopamine release in the “double-hit” PD mouse model (Figs. 1, 4), we evaluated mitochondrial function in DA neurons by quantifying cytosolic ATP/ADP dynamics using fiber photometry. We expressed the genetically encoded ratiometric ATP/ADP sensor PercevalHR [33] in SNc DA neurons by bilateral AAV9h-Syn-DIO-PercevalHR injection into *Dat*^IREScre/+^ mice (Fig. 5a, b). After four weeks of expression, optical fibers were implanted bilaterally in the SNc and spectrally resolved fiber photometry was performed using alternating 488-nm and 447-nm excitation to continuously monitor ATP/ADP *in vivo* (Fig. 5c).

During a 5-day NR treatment regimen (twice daily; 1000 mg/kg, i.p.), ATP/ADP levels were recorded before and after acute NR administration on days 1, 3, and 5. On day 1, NR did not significantly alter the ATP/ADP ratio (Fig. 5d). By contrast, on days 3 and 5, an acute NR injection elicited a strong increase in ATP levels accompanied by a reduction in ADP, resulting in a significantly elevated ATP/ADP ratio post-injection (Fig. 5e, f). These findings indicate that repeated NR treatment enhances mitochondrial energetic output in DA neurons.

### NR enhances mitochondrial energetics across striatal projection neuron subtypes

**Figure 6:**
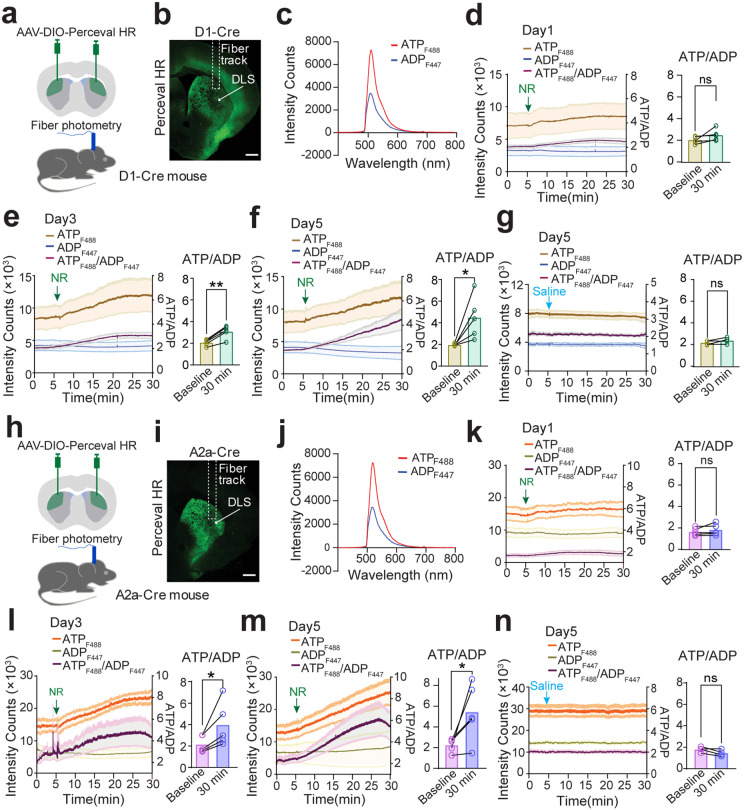
NR enhances mitochondrial energetics in SPNs **(a–c)** Expression of PercevalHR in D1-Cre mice via bilateral DLS injection of AAV9-hSyn-DIO-PercevalHR (a, b) and representative PercevalHR emission spectra excited by 488 nm (for detecting ATP) and 447 nm (for detecting ADP) lasers using spectrally resolved fiber photometry *in vivo* (c). **(d–g)** In dSPNs, NR did not alter ATP/ADP ratio significantly on day 1 (d) but produced robust elevations on days 3 and 5 of the twice-daily NR regimen (e-f). Saline did not alter ATP/ADP ratio following the same 5-day treatment regimen (g). Data are presented as mean ± SEM from 5 hemispheres of 3 mice/group (*n*=5) for NR or 4 hemispheres of 3 mice/group (*n*=4) for saline. Significance (*P < 0.05) was determined by paired t-test. **(h–j)** PercevalHR expression in A2A-Cre mice (h, i) and representative PercevalHR emission spectra excited by 488 nm (for detecting ATP) and 447 nm (for detecting ADP) lasers using spectrally resolved fiber photometry *in vivo* (j). **(k–n)** In iSPNs, NR produced no significant ATP/ADP change on day 1 (k), but significantly increased ATP/ADP ratios following NR administration on days 3 and 5 (l-m), mirroring effects seen in dSPNs. Saline did not alter ATP/ADP ratio following the same 5-day treatment regimen (n). Data are presented as mean ± SEM from 5 hemispheres of 3 mice/group (*n*=5) for NR or 4 hemispheres of 3 mice/group (*n*=4) for saline. Significance (*P < 0.05) was determined by paired t-test. **(c)** Summary statistics of consecutive quantification of total aldh1a1+ DA neurons in SNc and VTA. Data are presented as mean ± SEM from 6 mice/group (*n*=6). Significance (*P < 0.05) was determined by two-way ANOVA followed by Tukey’s multiple comparisons test. **(d)** Sequential quantification of aldh1a1+ terminal density expression in DLS. Data are presented as ± SEM from 4 mice/group. (*n*=4). Significance (*P < 0.05) was determined by two-way ANOVA followed by Tukey’s multiple comparisons test.

To determine whether NR supplementation enhances mitochondrial function beyond DA neurons, we next examined NR-induced ATP/ADP changes in striatal projection neurons (SPNs). We bilaterally injected AAV9-hSyn-DIO-PercevalHR into the DLS of D1-Cre and A2A-Cre mice to target direct- and indirect-pathway SPNs, respectively. After 4–5 weeks, allowing sufficient expression of the sensor, we implanted the fibers and performed fiber photometry recordings using the same NR administration protocol described above (Fig. 6a–c, h–g).

In D1-Cre mice, acute NR injection (1000 mg/kg, i.p.) did not alter the ATP/ADP ratio on day 1 (Fig. 6d). However, by days 3 and 5 of the 5-day twice-daily NR regimen, acute NR administration produced a robust increase in the ATP/ADP ratio in direct-pathway SPNs (dSPNs) (Fig. 6e, f). No such elevation occurred in saline-treated controls (Fig. 6g), confirming that the effect was NR-dependent.

Indirect-pathway SPNs (iSPNs) showed a similar pattern: A2A-Cre mice exhibited NR-induced increases in ATP/ADP ratio on days 3 and 5, but not on day 1 (Fig. 6k–m). Saline treated A2A controls exhibited no increase in ATP/ADP ratio (Fig. 6n). These findings indicate that NR-mediated enhancement of mitochondrial energetic output is not restricted to DA neurons but is likely a generalizable effect across multiple neuronal subtypes.

## Discussion

PD arises from a multifactorial interplay between genetic vulnerability, environmental exposures, and aging. To more accurately model this complexity, we developed a “double-hit” mouse model integrating α-synuclein (A53T) overexpression with chronic exposure to the ALDH inhibitor benomyl. This design was motivated by the “catecholaldehyde” hypothesis, which posits that toxic DOPAL accumulation and α-synuclein oligomerization drive DA vulnerability [40, 41]. Consistent with this framework, benomyl alone did not impair locomotion, whereas its combination with A53T α-synuclein produced progressive motor deficits, establishing a robust model of PD-like pathology.

We sought to identify metabolic interventions capable of slowing or compensating for PD-associated dysfunction. Nicotinamide riboside (NR), a precursor of NAD^+^, was selected due to its ability to enhance mitochondrial metabolism, support ALDH activity, and promote cellular resilience [42–44]. Strikingly, continuous NR supplementation significantly improved locomotion and motor coordination in the double-hit mice, despite not preventing DA neuron loss in the SNc, VTA, or their striatal projections. These findings indicate that NR primarily improves neuronal function rather than survival in this model.

Because striatal dopamine release tightly correlates with locomotor output [45, 46], we examined DA signaling in vivo. NR rapidly increased both tonic and evoked dopamine release in control mice and produced similar effects in double-hit PD mice, where dopamine release peaked within minutes of administration. Enhanced dopaminergic tone therefore provides a plausible substrate for the observed behavioral rescue.

ATP is a critical regulator of presynaptic dopaminergic function, and several complementary mechanisms likely underlie the NR-induced enhancement of dopamine release observed in our study. First, ATP is required for VMAT2-dependent vesicular dopamine loading, as the vesicular H^+^ gradient that drives dopamine uptake is generated by the ATP-dependent V-ATPase [47, 48]. ATP also sustains the Na^+^/K^+^-ATPase, which maintains membrane excitability and supports the Ca^2+^ influx necessary for activity-dependent vesicle fusion [49]. In addition, synaptic vesicle priming, docking, and recycling are energy-dependent processes that rely on ATP-driven SNARE complex regulation and actin remodeling [50]. DA terminals also depend on mitochondrial ATP production for local Ca^2+^ buffering, which directly shapes release probability and prevents presynaptic failure under metabolic stress [51]. Because SNc DA neurons possess extensive axonal arbors and exhibit autonomous pace-making activity, they operate near their energetic limits, making ATP availability a key determinant of dopamine release capacity [52, 53]. Thus, the NR-mediated increase in ATP/ADP ratios we observed likely enhances vesicular loading efficiency, supports presynaptic Ca^2+^ dynamics, and improves vesicle fusion probability, collectively contributing to the robust rise in tonic and evoked dopamine release in both control and double-hit PD mice.

Together, these findings identify NR as a metabolic modulator capable of restoring DA function without altering the trajectory of neuronal loss. Our results support the concept that boosting mitochondrial metabolism and enhancing residual DA output may represent a viable therapeutic strategy for mitigating PD symptoms. Future work will be required to determine how chronic NAD^+^ augmentation intersects with α-synuclein pathology, DOPAL detoxification, and long-term circuit adaptations in PD.

## Supplementary Material

Supplementary Files

This is a list of supplementary files associated with this preprint. Click to download.

- SuppNR.pdf

## Data Availability

All data generated or analyzed during this study are included in this published article. Source data are provided with this paper. Correspondence and requests for materials should be addressed to Huaibin Cai (caih@mail.nih.gov) or Guohong Cui (cuig@mail.nih.gov).
